# Fever-range hyperthermia improves the anti-apoptotic effect induced by low pH on human neutrophils promoting a proangiogenic profile

**DOI:** 10.1038/cddis.2016.337

**Published:** 2016-10-27

**Authors:** Fernando Erra Díaz, Ezequiel Dantas, Maia Cabrera, Constanza A Benítez, María V Delpino, Gabriel Duette, Julia Rubione, Norberto Sanjuan, Analía S Trevani, Jorge Geffner

**Affiliations:** 1 Instituto de Investigaciones Biomédicas en Retrovirus y SIDA (INBIRS), CONICET, Facultad de Medicina, Universidad de Buenos Aires, Buenos Aires, Argentina; 2 Instituto de Investigaciones Farmacológicas (ININFA), CONICET, Facultad de Farmacia y Bioquímica, Universidad de Buenos Aires, Buenos Aires, Argentina; 3 Instituto de Inmunología, Genética y Metabolismo (INIGEM), CONICET, Facultad de Farmacia y Bioquímica, Universidad de Buenos Aires, Buenos Aires, Argentina; 4 Instituto de Investigaciones en Microbiología y Parasitología Médica (IMPAM), CONICET, Facultad de Medicina, Universidad de Buenos Aires, Buenos Aires, Argentina; 5 Instituto de Medicina Experimental (IMEX), CONICET, Academia Nacional de Medicina, Buenos Aires, Argentina

## Abstract

Neutrophils have the shortest lifespan among leukocytes and usually die via apoptosis, limiting their deleterious potential. However, this tightly regulated cell death program can be modulated by pathogen-associated molecular patterns (PAMPs), danger-associated molecular pattern (DAMPs), and inflammatory cytokines. We have previously reported that low pH, a hallmark of inflammatory processes and solid tumors, moderately delays neutrophil apoptosis. Here we show that fever-range hyperthermia accelerates the rate of neutrophil apoptosis at neutral pH but markedly increases neutrophil survival induced by low pH. Interestingly, an opposite effect was observed in lymphocytes; hyperthermia plus low pH prevents lymphocyte activation and promotes the death of lymphocytes and lymphoid cell lines. Analysis of the mechanisms through which hyperthermia plus low pH increased neutrophil survival revealed that hyperthermia further decreases cytosolic pH induced by extracellular acidosis. The fact that two Na^+^/H^+^ exchanger inhibitors, 5-(N-ethyl-N-isopropyl) amiloride (EIPA) and amiloride, reproduced the effects induced by hyperthermia suggested that it prolongs neutrophil survival by inhibiting the Na^+^/H^+^ antiporter. The neutrophil anti-apoptotic effect induced by PAMPs, DAMPs, and inflammatory cytokines usually leads to the preservation of the major neutrophil effector functions such as phagocytosis and reactive oxygen species (ROS) production. In contrast, our data revealed that the anti-apoptotic effect induced by low pH and hyperthermia induced a functional profile characterized by a low phagocytic activity, an impairment in ROS production and a high ability to suppress T-cell activation and to produce the angiogenic factors VEGF, IL-8, and the matrix metallopeptidase 9 (MMP-9). These results suggest that acting together fever and local acidosis might drive the differentiation of neutrophils into a profile able to promote both cancer progression and tissue repair during the late phase of inflammation, two processes that are strongly dependent on the local production of angiogenic factors by infiltrating immune cells.

Neutrophils are rapidly recruited into sites of inflammation or infection to ingest and destroy microbes through the action of oxidants, proteases, and antimicrobial proteins. These toxic weapons do not discriminate self from non-self and cause bystander injury in the course of a variety of pathologic conditions.^1^ For this reason, the inflammatory activity of neutrophils is tightly regulated *in vivo* to avoid damage to the host. Apoptosis is the predominant cell death pathway in the neutrophil and represents one of the most important mechanisms responsible for its functional shutdown and the resolution of inflammation.^2^ Neutrophil apoptosis can be modulated by a variety of biological agents including cytokines, chemokines, lipid mediators, pathogen-associated molecular patterns (PAMPs), and danger-associated molecular patterns (DAMPs).^2, 3^ Less attention has been paid to the influence exerted by physicochemical alterations of the environment associated to inflammatory processes and cancer, such as local acidosis and fever-range hyperthermia.

Local acidosis (pH 5.5–7.0) is a hallmark of inflammatory conditions and solid tumors. Interstitial acidification is usually associated with the immune response against infectious agents in peripheral tissues.^4, 5^ Local acidosis is also a common feature of autoimmune and allergic diseases. pH values of 6.5 to 7.0 characterize the synovial fluid of compromised joints in patients with rheumatoid arthritis, with acidosis being associated to the accumulation of leukocytes and joint damage.^6, 7^ The pH of exhaled breath condensates from asthmatic patients is ∼5.2, whereas healthy subjects show values of 7.0 to 7.4.^8, 9^ Local acidosis is a major feature of solid tumors. Values of pH ranging from 5.5 to 7.0 are usually found in a variety of solid tumors such as adenocarcinomas, brain tumors, breast cancer, sarcomas, malignant melanoma, and squamous cell carcinomas.^10, 11, 12^ Interestingly, this acidic environment promotes tumor growth and metastasis.^13^ We have reported that low pH stimulates the activation of neutrophils^14^ and conventional dendritic cells.^15^ It also influences the function of monocytes, NK, and T cells.^16, 17, 18^ Moreover, we have previously reported that local acidosis moderately delays the rate of neutrophil apoptosis,^14^ and a similar anti-apoptotic effect was reported for endothelial and cancer cells.^19, 20^

The febrile response is one of the most commonly recognized features of inflammation and infection.^21^ Fever-range hyperthermia exerts a variety of immunomodulatory effects on immune cells including neutrophils.^21, 22^ It increases the number of circulating neutrophils as well as the recruitment of neutrophils to local sites of infection and tumors.^23^ Febrile temperatures modulate the production of reactive oxygen intermediates and nitric oxide by neutrophils,^24^ and accelerate the rate of neutrophil apoptosis.^25^ To our knowledge, no previous studies have examined the combined effect of low pH and fever-range hyperthermia on neutrophil survival and function. We here show that hyperthermia markedly increases neutrophil survival induced by low pH. Moreover, we found that febrile temperatures plus low pH induce the differentiation of neutrophils into a functional profile characterized by the paralysis of effector functions such as phagocytosis and reactive oxygen species (ROS) production, and a high ability to suppress T-cell activation and to produce angiogenic factors. This neutrophil profile might play a role in different processes such as cancer progression and tissue repair during the late phase of inflammation.

## Results

### Fever-range hyperthermia improves the anti-apoptotic effect induced by low pH on human neutrophils

**Figure 1:**
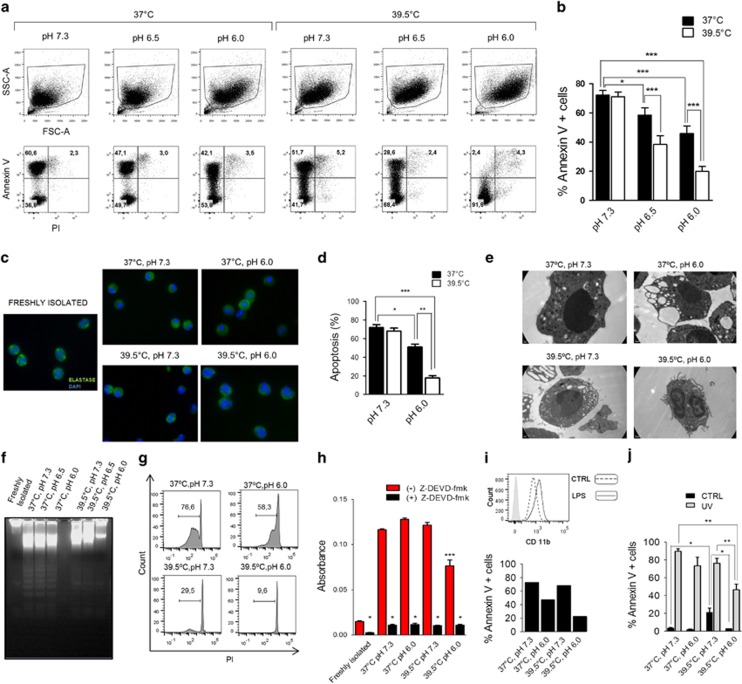
Fever-range hyperthermia enhances the neutrophil anti-apoptotic effect induced by low pH. (**a–h**) Neutrophils (1 × 10^6^/ml) were cultured at pH values of 7.3, 6.5, or 6.0 at 37 or 39.5 °C for 18 h. (**a** and **b**) Percentages of apoptotic cells were then evaluated by staining with annexin-V FITC/propidium iodide and flow cytometry. A representative experiment is shown in (**a**) and the mean±S.E. of nine experiments is shown in (**b**). (**c**) Morphological evaluation of apoptosis was performed by fluorescence microscopy in neutrophils labeled with a FITC-mAb directed to elastase and DAPI for nuclear acid staining. Representative images are shown. (**d**) Quantitation of neutrophil apoptosis was carried out by fluorescence microscopy using the fluorescent DNA-binding dyes acridine orange and ethidium bromide. Results are expressed as the mean±S.E. of five experiments. (**e**) Morphological evaluation of apoptosis was performed by electron microscopy. Representative images are shown. (**f–h**) DNA fragmentation assay (**f**), evaluation of cellular DNA content by propidium iodide staining and flow cytometry (**g**), and analysis of caspase 3 activity (**h**) were performed as described under Materials and Methods. Representative experiments are shown in (**f**) and (**g**), and the mean±S.E. of five experiments is shown (**h**). (**i**, upper panel) Neutrophils (1 × 10^6^/ml) were treated with LPS (200 ng/ml) for 1 h at 37 °C and pH 7.3, and the expression of CD11b, as a marker of neutrophil activation, was determined by flow cytometry. A representative experiment (*n*=4) is shown. (**i**, lower panel) Neutrophils (1 × 10^6^/ml) were treated with LPS (200 ng/ml) for 1 h at 37 °C and pH 7.3, washed and cultured for an additional period of 24 h at different pH (7.3 or 6.0) and temperatures (37 or 39.5 °C). Apoptosis was then evaluated by staining with annexin-V FITC/propidium iodide and flow cytometry. A representative experiment (*n*=4) is shown. (**j**) Neutrophils (1 × 10^6^/ml) were exposed, or not, to UV irradiation (254 nm) for 10 min. Then, cells were cultured for 8 h at different pH (7.3 or 6.0) and temperatures (37 °C or 39.5 °C) and apoptosis was evaluated by staining with annexin-V FITC/propidium iodide and flow cytometry. Data represent the mean±S.E. of four experiments. *P<0.05, **P<0.01, and ***P<0.001

We first analyzed the effect induced by low pH, hyperthermia, and low pH plus hyperthermia on neutrophil survival. Neutrophils were cultured at pH values of 7.3, 6.5, and 6.0 at 37 or 39.5 °C for 18 h and the percentages of apoptotic cells were then evaluated by staining with annexin-V FITC/propidium iodide and flow cytometry. Consistent with our previous results,^14^ we observed that low pH moderately delayed neutrophil apoptosis (Figures 1a and b). Hyperthermia did not induce any change in the rate of apoptosis at pH 7.3 but markedly improved the anti-apoptotic effect of low pH (Figures 1a and b). Similar results were observed when apoptosis was evaluated by fluorescence microscopy (Figures 1c and d), electron microscopy (Figure 1e), analysis of DNA fragmentation (Figure 1f), and analysis of cellular DNA content (Figure 1g). Consistent with these results, we observed that hyperthermia plus low pH significantly prevented the activation of caspase-3, an essential effector caspase of the extrinsic and intrinsic apoptotic pathways (Figure 1h).

The experiments described above were performed using resting neutrophils. We wondered whether a similar anti-apoptotic effect would be observed in previously activated neutrophils. In these experiments, neutrophils were treated with LPS for 1 h at 37 °C and pH 7.3, and washed and cultured for an additional period of 18 h at different pH (7.3 or 6.0) and temperatures (37 or 39.5 °C). Activation of neutrophils by LPS was confirmed by the enhancement in CD11b expression observed in neutrophils cultured with LPS for 1 h at 37 °C and pH 7.3 (Figure 1i, upper panel). As observed for resting neutrophils, we found that low pH plus hyperthermia markedly delayed the apoptotic rate of LPS-activated neutrophils (Figure 1i, lower panel). Of note, not only was the spontaneous rate of apoptosis delayed by the effect of low pH plus hyperthermia, but also the apoptotic rate of neutrophils exposed to ultraviolet irradiation (UV), a strong proapoptotic stimulus. Figure 1j shows that UV-treated neutrophils cultured at 37 °C and pH 7.3 displayed high levels of apoptosis (>80%) as early as at 8 h of culture, with the rate of apoptosis being significantly prevented in cell cultures performed at 39.5 °C and pH 6.0. In these experiments, and in agreement with a previous report,^25^ we also observed that untreated neutrophils cultured at 39.5 °C and pH 7.3 displayed an accelerated rate of apoptosis compared with cells cultured at 37 °C and pH 7.3 that was evident at 8 h (Figure 1j) but not at 18 h of culture (see Figures 1a and b). Interestingly, this early acceleration of apoptosis was completely prevented by low pH (Figure 1j).

**Figure 2:**
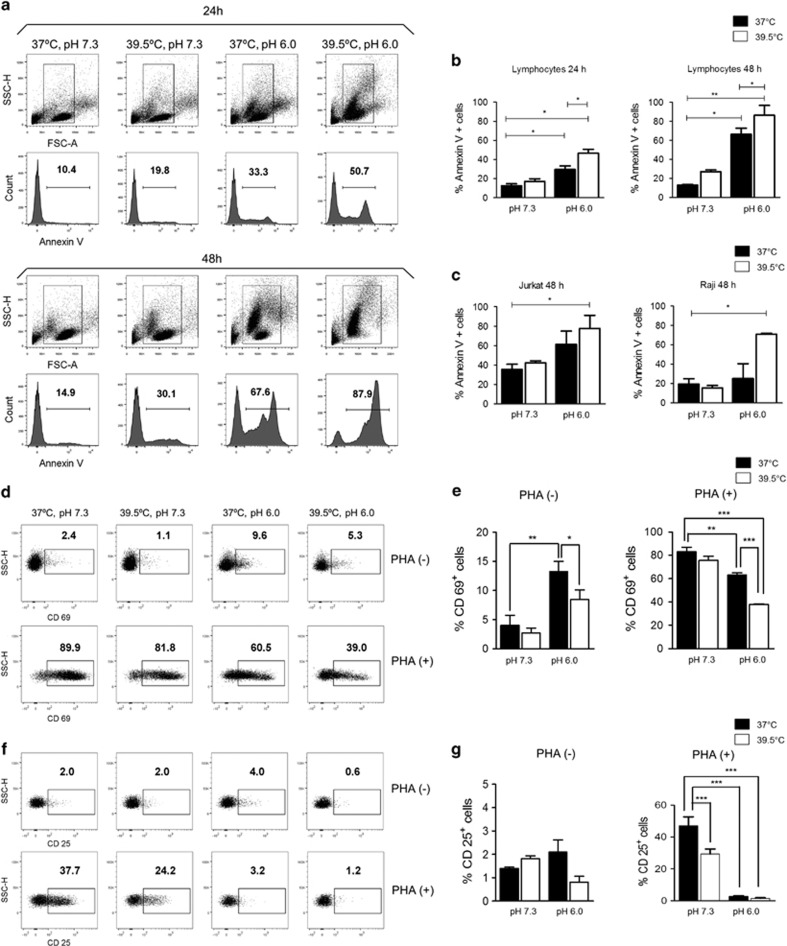
Fever-range hyperthermia enhances the proapoptotic effect of low pH on lymphocytes. PBMCs (1 × 10^6^/ml) were cultured at pH values of 7.3 or 6.0 at 37 or 39.5 °C for 24 or 48 h and the percentages of apoptotic cells were analyzed in the gate of lymphocytes by staining with annexin-V FITC/propidium iodide and flow cytometry. A representative experiment is shown in (**a**) and the mean±S.E. of six experiments is shown in (**b**). (**c**) The T-cell line Jurkat and the B-cell line Raji (3 × 10^5/^ml) were cultured for 48 h at pH values of 7.3 or 6.0 at 37 or 39.5 °C. Then, apoptosis was evaluated by staining with annexin-V FITC/propidium iodide and flow cytometry. Data represent the mean±SE of four experiments. (**d–g**) PBMCs (1 × 10^6^/ml) were cultured at pH values of 7.3 or 6.0 at 37 or 39.5 °C for 18 h with or without PHA (1 *μ*g/ml), and the expression of the activation markers CD69 (**d** and **e**) and CD25 (**f** and **g**) was analyzed by flow cytometry in the gate of viable lymphocytes. A representative experiment is shown in (**d**) and (**f**), and the mean±SE of four experiments is shown in (**e**) and (**g**). *P<0.05, **P<0.01, and ***P<0.001

To our best knowledge, no previous studies have analyzed the effect induced by low pH or hyperthermia on lymphocyte survival. Using peripheral blood mononuclear cells (PBMCs), we found that low pH and hyperthermia, acting individually, promoted lymphocyte death, with this proapoptotic effect being significantly increased when cells were simultaneously exposed to both conditions (Figures 2a and b). Similar results were observed using the B-cell line Raji and the T-cell line Jurkat (Figure 2c). We conclude that low pH plus hyperthermia induce opposite effects on the survival of neutrophils and lymphocytes. We next performed additional experiments to determine whether hyperthermia and acidosis were also capable of modulating lymphocyte activation. In these experiments, PBMCs were cultured for 18 h with or without PHA under different conditions of temperature and pH, and the expression of the activation markers CD69 and CD25 was analyzed in the gate of viable lymphocytes. We found that low pH (6.0) but not hyperthermia (39.5 °C) induced the expression of CD69 in a small fraction of lymphocytes (∼12%). In spite of this slightly stimulating effect, low pH and hyperthermia significantly prevented the expression of activation markers by PHA-stimulated lymphocytes (Figures 2d–g). We conclude that hyperthermia and low pH not only promote apoptosis, but also suppress lymphocyte activation.

### Analysis of the mechanisms through which hyperthermia improves the neutrophil anti-apoptotic effect induced by low pH

**Figure 3:**
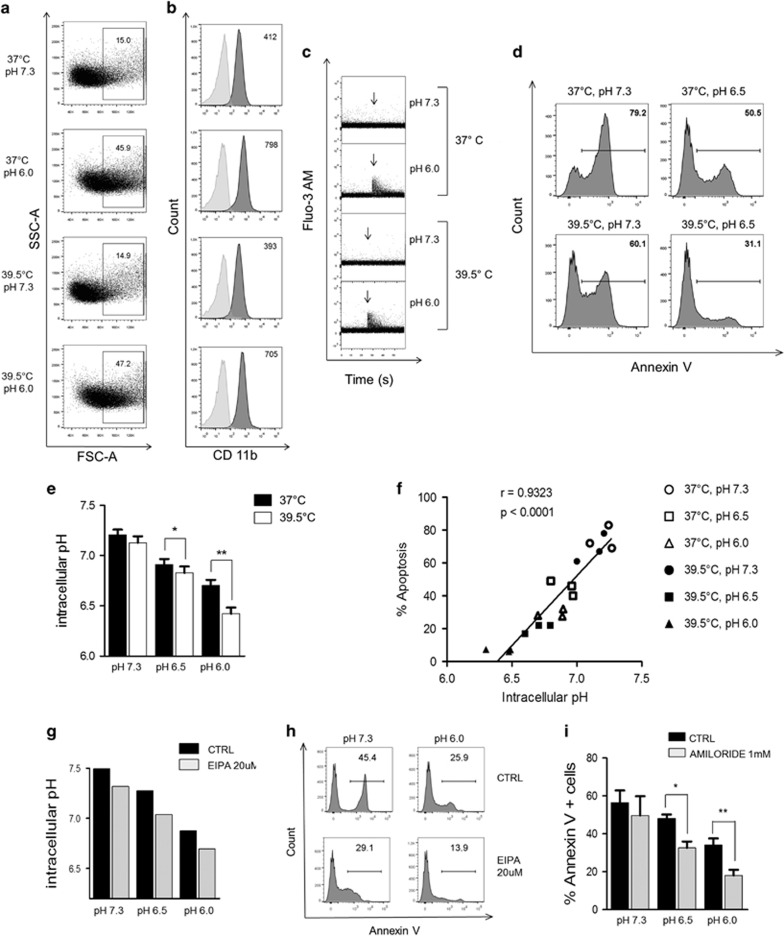
Analysis of the mechanisms through which hyperthermia enhances the neutrophil anti-apoptotic effect induced by low pH. (**a**) Neutrophils (1 × 10^6^/ml) were cultured for 10 min at 37 or 39.5 °C at pH 7.3 or pH 6.0, fixed by the addition of an equal volume of 2% glutaraldehyde, and the percentage of cells that had changed shape was evaluated by flow cytometry. A representative experiment (*n*=4) is shown. (**b**) Neutrophils (1 × 10^6^/ml) were cultured for 30 min at 37 or 39.5 °C at pH 7.3 or pH 6.0, and the expression of CD11b was analyzed by flow cytometry using a PE-labeled mAb directed to CD11b. A representative experiment (*n*=3) is shown. (**c**) The induction of cytosolic calcium transients in neutrophils cultured at 37 or 39.5 °C at pH 7.3 or pH 6.0 was evaluated using neutrophils labeled with fluo-3-AM and flow cytometry, as described under Materials and Methods. Arrows indicate the time at which PBS (pH 7.3) or an isotonic solution of HCl to lower the pH of the culture medium from 7.3 to 6.0 were added. A representative experiment (*n*=3) is shown. (**d**) Neutrophils (1 × 10^6^/ml) from a CGD patient were cultured at pH values of 7.3 or 6.5 at 37 or 39.5 °C for 24 h, and percentages of apoptosis were then evaluated by staining with annexin-V FITC and flow cytometry. A representative experiment from three independent experiments performed with different CGD patients is shown. (**e** and **f**) Neutrophils (1 × 10^6^/ml) were loaded, or not, with BCECF-AM (2 *μ*g/ml). After washing, cells were cultured at pH values of 7.3, 6.5, or 6.0 at 37 or 39.5 °C. After 4 h, cytosolic pH was determined as described under Materials and Methods, whereas apoptosis was evaluated after 18 h of culture by staining with annexin-V FITC/propidium iodide and flow cytometry. In (**e**), data represent the mean±S.E. of four experiments performed by duplicate. Panel (**f**) shows the positive correlation found between the values of cytosolic pH evaluated at 4 h of culture *versus* the percentages of apoptosis analyzed at 18 h of culture, for each experimental condition assessed. *P<0.01 and **P<0.001. (**g** and **h**) Neutrophils (1 × 10^6^/ml) were loaded, or not, with BCECF-AM (2 *μ*g/ml). After washing, cells were cultured at 37 °C and pH 7.3, 6.5, or 6.0 in the absence or presence of EIPA (20 *μ*M). Cytosolic pH was determined after 4 h of culture, whereas apoptosis was evaluated after 18 h of culture by staining with annexin-V FITC/propidium iodide and flow cytometry. A representative experiment (*n*=4) is shown. (**i**) Neutrophils (1 × 10^6^/ml) were cultured for 18 h at 37 °C at pH 7.3, 6.5, or 6.0 in the absence or presence of amiloride (1 mM). Apoptosis was then evaluated as described above. Data represent the mean±S.E. of four experiments. *P<0.05 and **P<0.01

The activation of neutrophils is usually associated to the induction of cytosolic Ca^2+^ transients and a delayed apoptotic rate.^3, 26^ We have previously reported that neutrophils cultured at low pH values undergo an increase in their forward light scattering properties, a rapid and transient enhance in cytosolic Ca^2+^, and the upregulation of CD11b expression.^14^ We speculated that hyperthermia might enhance the anti-apoptotic effect induced by low pH by further improving neutrophil activation. The experiments performed to test this hypothesis are shown in Figures 3a–c. We found that hyperthermia did not exert any effect on the early activation of neutrophils induced by low pH, measured as the increase in the neutrophil forward light scattering properties (Figure 3a), the upregulation of CD11b expression (Figure 3b), and the induction of Ca^2+^ transients (Figure 3c).

Because ROS are able to promote neutrophil apoptosis,^27^ and considering that low pH impairs the production of ROS by neutrophils,^28^ we wondered whether neutrophil survival induced by low pH plus hyperthermia could also be explained by a reduced production of ROS. To test this possibility, we used neutrophils isolated from chronic granulomatous disease (CGD) patients who are unable to produce ROS. Results in Figure 3d show that low pH plus hyperthermia delayed apoptosis of CGD neutrophils in a similar manner than control cells, suggesting that prolongation of neutrophil survival was not related to a decreased generation of ROS.

We have previously reported that lowering extracellular pH from 7.4 to 6.8 induces both a fall in intracellular pH (pHi) and a significant delay of neutrophil apoptosis,^14^ suggesting that extracellular acidosis might delay apoptosis by inducing a drop in pHi. Because previous studies have shown that hyperthermia promotes intracellular acidification in cancer cells,^29^ we hypothesized that hyperthermia could improve the prosurvival effect induced by low pH by lowering pHi. Results in Figure 3e show that hyperthermia did not decrease pHi of neutrophils cultured at pH 7.3, but significantly decreased pHi of neutrophils cultured at pH values of 6.5 and 6.0. Moreover, a close positive correlation was found between pHi and the rate of neutrophil apoptosis (Figure 3f). In order to establish a causal relationship between delayed apoptosis and intracellular acidification, we analyzed the effect of 5-(N-ethyl-N-isopropyl) amiloride (EIPA), an inhibitor of the most important proton extrusive mechanisms in the neutrophil, the Na^+^/H^+^ exchanger (NHE).^30^ EIPA decreased pHi of neutrophils cultured at pH values of 7.3, 6.5 or 6.0 and delayed neutrophil apoptosis (Figures 3g and h). Similar anti-apoptotic effects were observed using a second NHE inhibitor, amiloride (Figure 3i).

### Hyperthermia plus low pH leads to the paralysis of neutrophil effector functions and the acquisition of a proangiogenic profile

**Figure 4:**
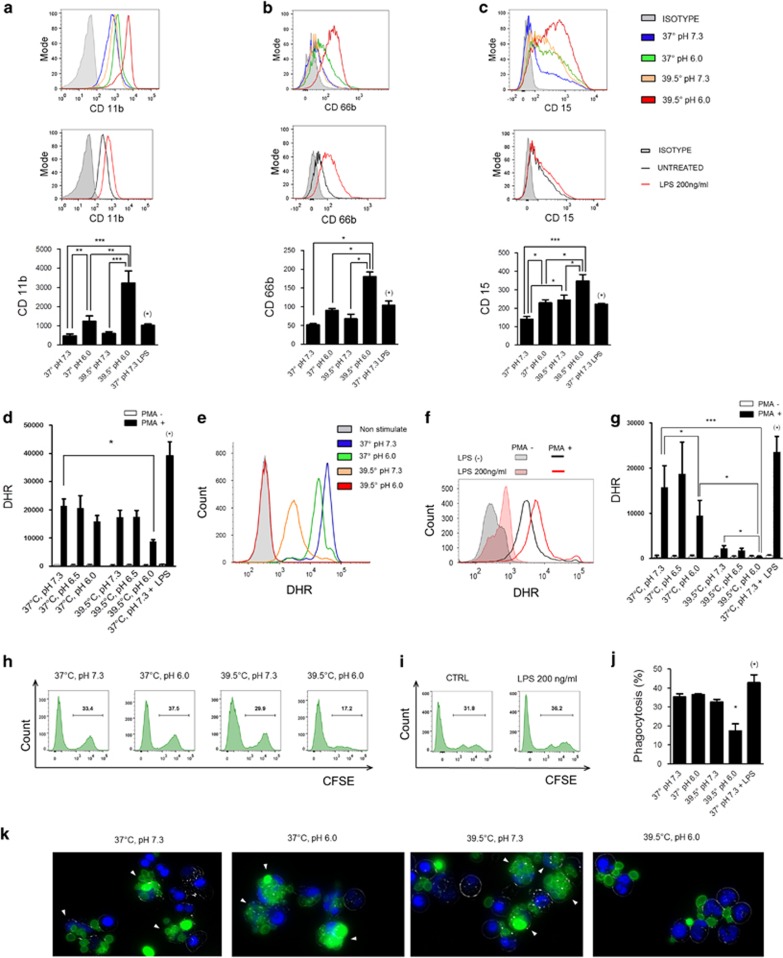
Analysis of the phenotype and effector functions of neutrophils exposed to low pH and fever-range hyperthermia. (**a–c**) Neutrophils (1 × 10^6^/ml) were cultured for 18 h at 37 or 39.5 °C at pH 7.3 or pH 6.0, and the expression of CD11b, CD66b, and CD15 was analyzed by flow cytometry excluding annexin-V-positive cells. The expressions of CD11b, CD66b, and CD15 by neutrophils (1 × 10^6^/ml) cultured with LPS (200 ng/ml) for 18 h at 37 °C and pH 7.3 are also shown. Representative experiments and the MFI (mean±S.E.) of 4–6 experiments are shown. (**d–g**) Neutrophils (1 × 10^6^/ml) were cultured for 4 h (**d**) or 18 h (**e** and **g**) at 37 or 39.5 °C at pH 7.3 or pH 6.0. In parallel experiments, neutrophils (1 × 10^6^/ml) were cultured with LPS (200 ng/ml) for 4 h (**d**) or 18 h (**f** and **g**) at 37 °C and pH 7.3. In all cases, after 4 or 18 h of culture, cells were washed, labeled with dihydrorhodamine-123 and stimulated with PMA (40 ng/ml), and the oxidation of dihydrorhodamine-123 to rhodamine-123 was analyzed by flow cytometry excluding annexin-V-positive cells. (**d** and **g**) Results represent the mean±S.E. of 4–7 experiments. (**e** and **f**) Representative experiments are shown. (**h** and **j**) Neutrophils (1 × 10^6^/ml) were cultured for 18 h at 37 or 39.5 °C at pH 7.3 or pH 6.0. In parallel experiments, neutrophils (1 × 10^6^/ml) were cultured with LPS (200 ng/ml) for 18 h (**i** and **j**). In all cases, cells were then washed and suspended in RPMI medium supplemented with 1% FCS at pH 7.3, and their ability to phagocyte CFSE-labeled *C. albicans* was assessed by flow cytometry (**h–j**) or fluorescence microscopy (**k**) using the neutrophil/*C. albicans* ratio of 1 : 5. Representative experiments (*n*=4–5) are shown in (**h**, **i**, and **k**) and the mean±S.E. of 5–8 experiments are shown in (**j**). *P<0.05, **P<0.01, and ***P<0.001. (*) *P*<0.05 *versus* 37 °C/pH 7.3

We then asked whether beside the ability to prolong neutrophil lifespan, hyperthermia plus low pH was able to change the functional profile of neutrophils. Neutrophils were cultured for 18 h (unless otherwise indicated) at 37 or 39.5 °C at pH values of 7.3, 6.5, or 6.0, and the phenotype and function of neutrophils were then analyzed by flow cytometry excluding annexin-V-positive cells. In these experiments, we also compared the effects induced by hyperthermia plus low pH with those induced by the traditional neutrophil agonist LPS. Results in Figures 4a and b show that the expression of the activation markers CD11b and CD66b was markedly upregulated in neutrophils cultured at pH 6.0 and 39.5 ºC. We also analyzed the expression of CD15 (Lewis^x^), a motif displayed on a number of surface glycoproteins such as CD18 and CD66 family members. Neutrophils cultured at pH 6.0 and 39.5 °C expressed higher levels of CD15 compared with those cells cultured at pH 7.3/37 ºC, pH 7.3/39.5 ºC, and pH 6.0/37 °C (Figure 4c). As expected, LPS-treated neutrophils (18 h at 37 °C/pH 7.3) also expressed higher levels of CD11b, CD66b, and CD15 over controls, but the expression of these three markers was significantly lower in LPS-treated neutrophils compared with cells cultured at pH 6.0 and 39.5 °C (*P*<0.05) (Figures 4a–c).

We then analyzed the ability of neutrophils to produce ROS. Of note, neutrophils cultured at low pH and 39.5 °C for 4 h (Figure 4d) and 18 h (Figures 4e and g) progressively lost their ability to produce ROS in response to PMA stimulation. Similar results were observed using the chemotactic peptide fMLP as stimulus (not shown). It should be noted that neutrophils cultured at 39.5 °C and pH 7.3 for 18 h, but not for 4 h, also showed a marked reduction in their ability to produce ROS, indicating that the sole exposure to hyperthermia for long periods impairs the subsequent activation of the neutrophil oxidative burst. As expected, these results contrast with those observed in LPS-treated neutrophils. Consistent with the known ability of LPS to prime the neutrophil oxidative burst,^31^ we found that LPS treatment increased ROS production by PMA-activated neutrophils (Figures 4d, f and g). We then analyzed the ability of neutrophils to phagocyte *Candida albicans* by flow cytometry. Neutrophils cultured for 18 h at 39.5 °C and pH 6.0 displayed a low phagocytic activity (Figures 4h and j). In fact, analysis of phagocytosis by fluorescence microscopy revealed that neutrophils cultured at 39.5 °C and pH 6.0 were completely unable to ingest yeast particles (Figure 4k). In contrast, LPS treatment induced a low but significant increase in the ability of neutrophils to ingest *C. albicans* (Figures 4i and j). We conclude that exposure to febrile-range temperatures and low pH leads to a progressive paralysis of the major neutrophil effector functions.

**Figure 5:**
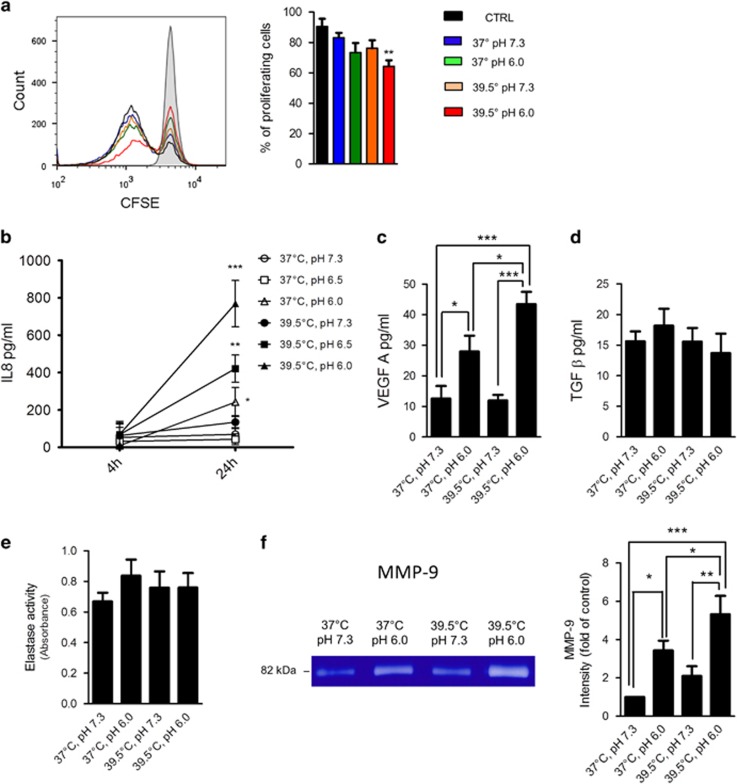
Exposure to low pH plus hyperthermia enhances the ability of neutrophils to suppress T-cell proliferation and to produce IL-8, VEGF, and MMP-9. (**a**) Neutrophils (1 × 10^6^/ml) were cultured for 18 h at 37 or 39.5 °C at pH 7.3 or pH 6.0. Cell supernatants were then harvested, adjusted to pH 7.3, and their ability to modulate the proliferation of autologous T cells labeled with CFSE, induced by PHA (1 *μ*g/ml), was assessed as described under Materials and Methods. Histograms illustrate a representative experiment, and bars represent the mean±S.E. of four experiments. **P<0.01 *versus* controls. (**b–e**) Neutrophils (1 × 10^6^/ml) were cultured for 4 h (only in (**b**)) or 18 h at 37 or 39.5 °C at pH 7.3, 6.5, or 6.0, and the concentration of IL-8, VEGF, TGF-*β*, and the activity of elastase were quantified as described in Materials and Methods. Data represent the mean±S.E. of 4–7 experiments. In (**b**), ***P<0.001 *versus* 37 ºC/pH 7.3, 37 ºC/pH 6.0; **P<0.05 *versus* 37 ºC/pH 7.3 and 37 ºC/pH 6.0; *P<0.05 *versus* 37 ºC/pH 7.3. In (**c**), *P<0.05, and ***P<0.001. (**f**) Neutrophils (1 × 10^6^/ml) were cultured for 18 h at 37 or 39.5 °C at pH 7.3 or pH 6.0. Cell supernatants were then harvested, adjusted to pH 7.3, and gelatinase activity was assessed as described under Materials and Methods. Densitometric results are expressed as arbitrary units as the mean±S.E. of four experiments. *P<0.05, **P<0.01, and ***P<0.001

It has become clear in the past years that the function of neutrophils is not restricted to the phagocytosis and killing of internalized pathogens. Neutrophils are not only able to produce a variety of cytokines such as IL-1*β*, TNF-*α*, IL-6, IL-12p70, IL-8, and VEGF-*β*, but are also capable of modulating the course of adaptive immunity.^32^ We analyzed the ability of neutrophils to modulate the activation of T cells. Figure 5a shows that supernatants from neutrophils cultured at pH 6.0 and 39.5 ºC, but not those harvested from neutrophils cultured at pH 7.3/37 ºC, pH 7.3/39.5 ºC, and pH 6.0/37 ºC, suppressed the proliferative response of T cells induced by PHA. We next examined the ability of neutrophils to produce different cytokines. No significant production of IL-1, IL-6, TNF-*α*, or IL-10 was detected in any of the experimental conditions analyzed. In contrast, we found that low pH significantly stimulated the production of IL-8 and VEGF, with this production being further enhanced in neutrophils cultured at low pH values and 39.5 °C (Figures 5b and c). No changes were observed in the release of either TGF-*β* or the serine proteinase elastase (Figures 5d and e). Because neutrophils are one of the most important sources of the matrix metalloproteinase-9 (MMP-9), a potent proangiogenic factor,^33^ we also analyzed the production of this enzyme. Figure 5f shows that pH 6.0 significantly stimulated the release of MMP-9, with this response being further enhanced in neutrophils cultured at pH 6.0 and 39.5 °C. Together, these results suggest that exposure of neutrophils to fever-range hyperthermia and low pH promotes the acquisition of a proangiogenic profile.

## Discussion

In this study, we show that febrile-range hyperthermia markedly enhanced the neutrophil anti-apoptotic effect induced by low pH. This effect was unexpected because hyperthermia accelerated the rate of neutrophil apoptosis at pH 7.3, as previously described.^25^ Considering previous studies showing that fever-range hyperthermia decreases cytosolic pH in cancer cells,^29^ we hypothesized that hyperthermia might delay apoptosis of neutrophils cultured at low pH by further lowering cytosolic pH. Our results supported this hypothesis. Moreover, we found that two Na^+^/H^+^ exchanger inhibitors, EIPA and amiloride, mimic the effect of febrile-range temperature, decreasing cytosolic pH and delaying neutrophil apoptosis in cultures performed at low pH.

Interestingly, and contrasting with the observations made in neutrophils, we found that low pH accelerated the rate of lymphocyte apoptosis and this proapoptotic response was further enhanced when lymphocytes were simultaneously exposed to low pH and 39.5 ºC. This interesting paradox might reflect some unique molecular features of the neutrophil such as a very short half-life, a reduced number of functional mitochondria, their ability to generate huge concentrations of ROS through the activation of the NADPH oxidase, and the presence of a high concentration of proteases in their granules.^34^ In fact, previous studies have shown important differences in the regulation of apoptosis between neutrophils and lymphocytes. For instance, induction of cytosolic calcium transients accelerates the rate of apoptosis of thymocytes, mature T cells, B lymphocytes, and leukemic T-cell lines.^35, 36, 37^ In contrast, elevation of cytosolic calcium delays neutrophil apoptosis.^26^ In the same line of thought, cytosolic acidification has shown to promote the apoptosis of T^38^ and B lymphocytes,^39^ whereas our present results and those previously described by Kaba *et al.*^40^ indicate that a decreased cytosolic pH delays neutrophil apoptosis.

In a previous study, Nagarsekar *et al.*^25^ showed that febrile-range hyperthermia (39.5 ºC) accelerated the rate of apoptosis in resting neutrophils. They reported that almost 90% of neutrophils displayed apoptotic features after 8 h of culture at 39.5 ºC, with the levels of apoptosis being lower than 20% for neutrophils cultured at 37 ºC. In agreement with this observation, we found that febrile-range hyperthermia accelerated the early rate of apoptosis: 4 *versus* 20% of apoptotic cells for neutrophils cultured during 8 h at 37 °C and 39.5 °C, respectively (Figure 1j). However, we did not found any differences between the percentages of apoptotic cells when they were evaluated at 24 h of culture, the time at which the percentages of apoptosis reach values between 60 and 80% for cells incubated at either 37 °C or 39.5 ºC. Our results show quantitative rather than qualitative differences with those reported by Nagarsekar *et al.*^25^ They could be explained, at least in part, by differences in the experimental conditions used, such as the source and the concentration of serum in the culture medium, the procedures employed to purify neutrophils, and the concentration of neutrophils in cell cultures. Beyond these differences, our observations suggest that neutrophils contain a subpopulation of cells highly sensitive to febrile-range hyperthermia and reinforce the notion that peripheral blood neutrophils do not represent a homogeneous cell population. Interestingly, previous studies directed to characterize the ability of TNF-*α* to modulate neutrophil apoptosis have also shown a heterogeneous susceptibility to early induction of apoptosis. In fact, TNF-*α* has been variably reported to either delay, induce, or have no effect on neutrophil apoptosis when it was evaluated after 18–24 h of culture;^41, 42^ however, kinetic studies revealed that TNF-*α* consistently accelerated apoptosis in a subpopulation of neutrophils in short-term cultures (2 to 8 h).^41^

It is now widely recognized that once recruited in peripheral tissues, neutrophils may display a remarkable plasticity.^1, 43^ Our present results reveal that prolongation of neutrophil survival induced by low pH plus fever-range hyperthermia results in the induction of a functional profile characterized by the paralysis of the effector functions such as ROS production and phagocytosis, together with a high ability to prevent T-cell activation and to produce the proangiogenic factors VEGF, IL-8, and MMP-9. Interestingly, some of these functional and phenotypic features resemble those seen in sepsis.^44, 45^ In contrast with lymphocytes, which show an accelerated rate of apoptosis, neutrophil apoptosis is delayed in the course of sepsis.^44, 45^ Moreover, a reduced generation of ROS, an enhanced production of IL-8, and a high expression of CD11b and CD66b are usually found in neutrophils from septic patients.^44, 45^

A large body of evidence supports the notion that a high numbers of tumor-infiltrating neutrophils predict a poor clinical outcome in cancer patients, and current evidence strongly suggests that infiltrating neutrophils promote cancer progression primarily by stimulating tumor angiogenesis.^43, 46^ VEGF, IL-8, and MMP-9 are three of the most important factors responsible for tumor angiogenesis,^47^ and our present results indicate that under the influence of low pH and fever-range hyperthermia, neutrophils markedly increase their ability to release these proangiogenic factors. VEGF is a potent angiogenic factor and a validated therapeutic target for inhibiting tumor progression.^48^ Of note, tumor-associated neutrophils appear to be the major source of MMP-9 in the tumor microenvironment.^49^ This enzyme plays an important role in tumor angiogenesis by remodeling the extracellular matrix, releasing thereby activating bound growth factors such as VEGF.^49^ Moreover, it has been shown that MMP-9 processes the neutrophil chemoattractant and angiogenic chemokine IL-8, markedly increasing its biological activity.^50^ Together, these observations suggest that VEGF, MMP-9, and IL-8 could act synergistically to promote both angiogenesis and cancer progression. Neutrophils are also able to produce other angiogenic factors such as prokinecitin-2 (PK2), also known as Bv8, that appears to play an important role not only in tumor angiogenesis, but also in tumor refractoriness to anti-VEGF treatments.^51, 52^ Whether low pH plus fever-range hyperthermia might also be able to modulate neutrophil production of PK2/Bv8 remains to be determined.

Interestingly, our present results reveal that neutrophils cultured at low pH and normothermia also acquire a proangiogenic profile, although it was less pronounced compared with neutrophil cultured at low pH and febrile-range temperature. This observation supports a role for extracellular pH as a master regulator of the proangiogenic activity of neutrophils. In contrast, neutrophils cultured under hyperthermia and normal pH fail to acquire a proangiogenic profile, but progressively lose their ability to produce ROS. This condition (i.e., hyperthermia and normal pH) might occur in a number of situations. For example, during the course of localized bacterial infections, the pools of bone marrow and circulating neutrophils are likely to be faced to hyperthermia at neutral pH values. Further studies are required to determine whether febrile-range temperatures actually compromise *in vivo* the neutrophil microbicidal activity.

The factors responsible for the differentiation of tumor-infiltrating neutrophils into a proangiogenic profile have not yet been defined. Our results suggest that the angiogenic switch represents the most prominent change in the functional profile of neutrophils induced by fever-range hyperthermia and low pH. Because extracellular acidosis (pH 5.5–7.0) is a hallmark of solid tumors,^10, 11, 12, 13^ our results suggest that low pH might play an important role in the acquisition of a proangiogenic profile by tumor-infiltrating neutrophils.

## Materials and Methods

### Reagents

Acridine orange, ethidium bromide, fluo-3AM, BCECF-AM (AM (2',7'-bis-(2-carboxyethyl)-5-(and-6)-carboxyfluorescein, acetoxymethyl ester), dihydrorhodamine 123, carboxyfluorescein succinimidyl ester (CFSE), amiloride, and EIPA were obtained from Sigma-Aldrich (St. Louis, MO, USA). The cell lines Jurkat and Raji were obtained from the AIDS Research and Reference Reagent program (AIDS Division, National Institute of Allergy and Infectious Disease, National Institutes of Health, Bethesda, MD, USA).

### Isolation of neutrophils and PBMCs

The studies performed in this work have been reviewed and approved by the institutional review board and local ethical committee. Human subjects gave written informed consent. Neutrophils were isolated from heparinized human blood samples collected from healthy donors or X-CGD patients by centrifugation on Ficoll-Paque (GE Healthcare, Argentine) and dextran (Sigma-Aldrich) sedimentation. Contaminating erythrocytes were removed by hypotonic lysis. After washing, the cell pellets (>98% of neutrophils on May-Grunwald-Giemsa-stained cytopreparations) were suspended in RPMI-1640 medium (Gibco Invitrogen, Carlsbad, CA, USA) supplemented with 1% heat-inactivated fetal calf serum (FCS), 50 U/ml penicillin, and 50 *μ*g/ml streptomycin (Gibco Invitrogen). PBMCs were isolated from the interface of the Ficoll-Paque gradient, washed and suspended in RPMI-1640 medium supplemented with 10% FCS, 50 *U*/ml penicillin, and 50 *μ*g/ml streptomycin.

### Culture conditions

Neutrophils (1 × 10^6^/ml) were cultured for different periods at 37 or 39.5 °C in 5% CO_2_ at pH values of 7.3, 6.5, and 6.0 in RPMI medium supplemented with 1% FCS. LPS-treated neutrophils were obtained by neutrophil (1 × 10^6^/ml) treatment with 200 ng/ml of LPS from *Escherichia coli* (Sigma-Aldrich) for 18 h at 37 °C and pH 7.3. PBMCs (1 × 10^6^/ml) were cultured for different periods at 37 or 39.5 °C in 5% CO_2_ at pH values of 7.3 and 6.0 in RPMI medium supplemented with 10% FCS. The T-cell line Jurkat and the B-cell line Raji were cultured for 48 h in RPMI medium supplemented with 1% FCS. Extracellular acidification was achieved by suspending cell pellets in culture medium previously adjusted to the desired pH values by the addition of distinct volumes of an isotonic hydrogen chloride solution, as we previously described.^14, 53^

### Quantitation of neutrophil apoptosis by annexin-V binding and flow cytometry

Annexin-V binding to apoptotic neutrophils was carried out using an apoptosis detection kit (Immunotech, Argentine). Cells were labeled with annexin-V FITC and propidium iodide for 15 min at 4 °C and analyzed by flow cytometry using a BD FACSCanto cytometer and BD FACSDiva software (BD Biosciences, Franklin Lakes, NJ, USA).

### Evaluation of cellular apoptosis by fluorescence microscopy

Morphological evaluation of apoptosis was performed in neutrophils labeled with Alexa 488-mAb directed to elastase and DAPI (4',6-diamidino-2-phenylindole) for nuclear acid staining. Quantitation of neutrophil apoptosis was carried out as previously described^54^ using the fluorescent DNA-binding dyes acridine orange (100 *μ*g/ml) to determine the percentage of apoptotic cells and ethidium bromide (100 *μ*g/ml) to differentiate viable from nonviable cells. With this procedure, nonapoptotic cell nuclei show variations in fluorescence intensity reflecting the distribution of euchromatin and heterochromatin. In contrast, apoptotic nuclei show highly condensed chromatin that is uniformly labeled by acridine orange. To determine the percentage of apoptotic cells, at least 200 cells were analyzed in each experiment. Previous reports have shown that the results obtained by this method closely correlates with those obtained using other methods to evaluate apoptosis, such as propidium iodide staining and annexin-V binding.^54^

### DNA fragmentation assay

DNA fragmentation assay was performed as previously described.^55^ Briefly, 100 *μ*l of DMSO was added directly to the cell pellet and mixed followed immediately by vortexing. Equal volume (100 *μ*l) of TE buffer (pH 7.4) with 2% SDS was added, followed by mixing and vortexing. Samples were analyzed by gel electrophoresis (agarose 1.8%) and staining with ethidium bromide. The gels were examined under UV transillumination.

### Evaluation of cellular DNA content by propidium iodide staining and flow cytometry

The frequency of neutrophils that show a hypodiploid DNA peak was evaluated as previously described.^56^ Briefly, cell pellets containing 2.5 × 10^6^ neutrophils were resuspended in 0.4 ml of hypotonic fluorochrome solution (50 *μ*g/ml of propidium iodide in 0.1% sodium citrate plus 0.1% Triton X-100) and incubated for 2 h at 4 °C. The red fluorescence of propidium iodide in individual nuclei was evaluated using a using a BD FACSCanto cytometer and BD FACSDiva software (BD Biosciences).

### Neutrophil morphology evaluated by electron microscopy

Studies were performed using a transmission electron microscope (1011; JEOL, Pleasanton, CA, USA) at 60 kV. Images were recorded with a cooled charge-coupled device digital camera (MegaView III; Olympus, Center Valley, PA, USA).

### Calcium measurements

Changes in intracellular concentrations of free calcium ([Ca^2+^]_i_) were analyzed using fluo-3-AM (BD Pharmingen, Waltham, MA, USA), as previously described.^57^ Neutrophils, at a concentration of 2.5 × 10^6^ cells/ml, were treated with 4 *μ*M fluo-3-AM for 30 min at 30 °C. Then, cells were washed and resuspended at 5.0 × 10^6^ cells/ml in RPMI-1640 supplemented with 5% FCS. Aliquots of this cellular suspension (50 *μ*l) were added to 400 *μ*l ml of RPMI-1640 medium supplemented with 5% FCS, prewarmed at 37 or 39.5 °C. The prewarmed sample was immediately loaded onto the flow cytometer, and fluorescence was evaluated for ∼20 s. After this time, the medium was acidified, or not, by the addition of a predetermined volume of isotonic solution of HCl to adjust its pH to 6.0, and the fluorescence was recorded during an additional period of 100 s. A time-based gate was used to divide the original data file and separate cells according to the time at which their fluorescence in an FL1 detector was measured. The fluorescence of samples suspended at pH 7.3 and 37 °C were used to establish a marker at a FL1 fluorescence channel number greater than that exhibited by at least 97% of these resting cells. This marker was then used to determine the relative percentage of activated cells. Cells that raised their [Ca^2+^]_i_ to higher levels than that shown by 97% of resting cells in response to pH 6.0 and/or hyperthermia were considered to be activated.

### Neutrophil shape change

Neutrophils (1 × 10^6^/ml in RPMI-1640 medium with 1% FCS) were incubated for 10 min at 37 or 39.5 °C at pH 7.3 or pH 6.0, fixed by the addition of glutaraldehyde, at a final concentration of 2%, and the cell shape change was then evaluated by flow cytometry as the shift in the forward light scatter parameter.^14^

### Measurement of pH_i_

Measurement of pHi was performed using BCECF-AM as previously described.^58^ Neutrophils (1 × 10^6^/ml in PBS) were loaded with 2 *μ*g/ml BCECF-AM during 15 min at 37 °C, washed in PBS, and resuspended in culture medium (1 × 10^6^ /ml) adjusted to the different pH values. Cells were incubated for 4 h, in the absence or presence of 20 *μ*M EIPA at 37 or 39.5 ºC, and the values of pHi for each condition were determined. Analysis was performed by flow cytometry, with excitation at 488 nm and emission analysis at FL1 and FL3. The pHi was calculated from the ratio of emission intensities at the two wavelengths, standardizing by comparison with the fluorescence intensity ratios of cells whose pHi values were fixed by incubation with nigericin (10 *μ*M) in high-potassium buffers.

### Flow cytometric determination of hydrogen peroxide production

Generation of hydrogen peroxide was assessed by quantifying the intracellular oxidation of the indicator dye dihydrorhodamine-123 (Molecular Probes, Carlsbad, CA, USA), a membrane-permeable fluorogenic substrate that is oxidized by hydrogen peroxide to the fluorescent compound rhodamine-123.^59^ Briefly, neutrophils (1 × 10^6^/ml) were cultured for 4 or 18 h at 37 or 39.5 °C at pH 7.3 or pH 6.0. Then, cells were washed, resuspended at pH 7.3, labeled with dihydrorhodamine-123, and stimulated with PMA (40 ng/ml). The oxidation of dihydrorhodamine-123 to rhodamine-123 was analyzed by flow cytometry, using a BD FACSCanto cytometer and BD FACSDiva software. Control experiments were performed in order to establish that the uptake of dihydrorhodamine-123 was similar for neutrophils previously cultured for 18 h at 37 or 39 °C at pH values of 7.3 or 6.0. We observed that all neutrophil preparations reached similar mean fluorescence intensity values upon the addition of exogenous hydrogen peroxide (50 *μ*M) (data not shown).

### Phagocytosis of *C. albicans*

*Ca. albicans* was grown to stationary phase in YPD medium (Sigma-Aldrich) at 30 °C with orbital shaking at 160 r.p.m. Labeling of *C. albicans* with CFSE (Invitrogen, Carlsbad, CA, USA) was performed by incubating 1 × 10^8^ yeasts with CFSE (0.5 *μ*mol in 1 ml PBS) for 1 h at 37 °C. Yeast cells were then washed twice in PBS and phagocyte assays were performed by incubating neutrophils and CFSE-labeled yeasts at a neutrophil/yeast ratio of 1 : 5, during 45 min at 37 °C. Phagocytosis was then evaluated by flow cytometry or fluorescence microscopy.

### Caspase 3 activity assay

Neutrophils (1 × 10^6^/ml) were cultured for 18 h at 37 or 39.5 °C in 5% CO_2_ at pH values of 7.3, 6.5, and 6.0. Cells were then harvested and processed according to the caspase 3 colorimetric assay kit (Sigma-Aldrich). Absorbance due to the hydrolysis of the peptide substrate acetyl-Asp-Glu-Val-Asp p-nitroanilide (Ac-DEVD-pNA) was measured at 405 nm using the FlexStation 3 microplate reader (Molecular Devices Inc., Sunnyvale, CA, USA), and caspase 3 activity was expressed as OD_405_ values.

### Analysis of neutrophil phenotype by flow cytometry

FITC- or PE-labeled mAbs directed to CD11b, CD15, and CD66b were obtained from BD Biosciences. Analysis was performed by using a BD FACSCanto cytometer and BD FACSDiva software.

### Lymphocyte activation assays

PBMCs (1 × 10^6^/ml) were cultured in 48-well plates in RPMI-1640 medium supplemented with 10% FCS for 18 h under different conditions of temperature and pH, in the absence or presence of PHA (1 *μ*g/ml). Then, the expression of CD69 and CD25 was analyzed by flow cytometry in the gate of viable lymphocytes, using specific FITC-labeled mAbs (BD Biosciences).

### Lymphocyte proliferation assays

PBMCs were diluted in PBS (1 × 10^7^ cells/ml) and incubated with an equal volume of 2 *μ*M CFSE (Molecular Probes, Invitrogen) in PBS for 10 min at 37 °C. Cells were washed and plated at a final density of 1 × 10^6^ cells/ml in 48-well plates in fresh RPMI-1640 medium supplemented with 10% FCS and 20 mM HEPES. Cells were cultured with or without supernatants (50% V/V) collected from autologous neutrophils (1 × 10^6^/ml) incubated for 18 h at 37 or 39 °C and pH 7.3 or 6.0, previously adjusted to pH 7.3. Then, PBMCs were stimulated by PHA (1 *μ*g/ml) for 3 days, and cell proliferation was assessed by flow cytometry.

### Analysis of elastase activity

Elastase activity in neutrophil supernatants was measured using the specific chromogenic substrate Glp-Pro-Val-p-nitroanilide (Sigma-Aldrich), by measuring absorbance at 405 nm.

### Zymography

Gelatinase activity was assayed as previously described.^60^ Briefly, 50 *μ*l of neutrophil supernatants (1 × 10^6^ cells/ml) were mixed with 10 *μ*l of 5 × loading buffer (0.25 M Tris pH 6.8, 50% glycerol, 5% SDS, and bromophenol blue crystals) and loaded onto 10% SDS-PAGE gels containing 1 mg/ml gelatin (Sigma-Aldrich). Following electrophoresis, gels were washed using a solution with 50 mM Tris-HCl, pH 7.5, and 2.5% Triton X-100 (buffer A) for 30 min. Then, gels were washed with buffer A supplemented with 5 mM CaCl_2_ and 1 *μ*M ZnCl_2_ for 30 min, and incubated with buffer A supplemented with 10 mM CaCl_2_ and 200 mM NaCl for 48 h at 37 °C, as previously described.^60^ Gelatinase activity was revealed by staining with 0.5% Coomassie blue. The unstained band indicated the presence of gelatinase activity, and the band position indicated the molecular weights of the enzyme. Densitometric results are expressed as arbitrary units.

### Measurement of cytokines by ELISA

Neutrophils (1 × 10^6^/ml) were cultured for different periods at 37 or 39.5 °C in 5% CO_2_ at pH values of 7.3, 6.5, and 6.0. Then, supernatants were harvested and analyzed for the presence of IL-1, TNF-*α*, IL-6, IL-8, IL-10, VEGF, and TGF-*β* by ELISA, according to the manufacturer's instructions (R&D Systems, Minneapolis, MS, USA).

### Statistical analysis

Data were analyzed using Wilcoxon nonparametric paired test in which *P-*values of <0.05 were considered statistically significant.

## Abbreviations

MMP-9: matrix metallopeptidase 9
ROS: reactive oxygen species
PAMP: pathogen-associated molecular pattern
DAMPs: danger-associated molecular pattern
NHE: Na^+^/H^+^ exchanger
EIPA: 5-(N-ethyl-N-isopropyl) amiloride
BCECF-AM: AM (2',7'-bis-(2-carboxyethyl)-5-(and-6)-carboxyfluorescein, acetoxymethyl ester
CFSE: carboxyfluorescein succinimidyl ester
CGD: chronic granulomatous disease
PBMC: peripheral blood mononuclear cell
